# Prevention, diagnosis and management of community acquired respiratory virus infections including COVID-19 in patients with cancer: 2025 updated evidence-based guideline of the infectious diseases working party (AGIHO) of the German society for hematology and medical oncology (DGHO)

**DOI:** 10.1007/s00277-026-06769-9

**Published:** 2026-01-21

**Authors:** Nicola Giesen, Sibylle C. Mellinghoff, Elham Khatamzas, Felix Korell, Marcus Hentrich, Hermann Einsele, Larissa Henze, Claus Peter Heußel, Christian Hohmann, Björn-Erik Ole Jensen, Malte B. Monin, Philippe Schafhausen, Enrico Schalk, Karsten Spiekermann, Sebastian Voigt, Marie von Lilienfeld-Toal, Daniel Teschner, Oliver A. Cornely, Christina Rieger, Elena Busch

**Affiliations:** 1 Department of Hematology, Oncology and Palliative Care, Robert Bosch Hospital, Auerbachstr. 110, Stuttgart, 70376 Germany; 2 Robert Bosch Center for Tumor Diseases (RBCT), Stuttgart, Germany; 3 Department I of Internal Medicine, Medical Faculty and University Hospital Cologne, University of Cologne, Cologne, Germany; 4 Institute of Translational Research, Cologne Excellence Cluster on Cellular Stress Responses in Aging-Associated Diseases (CECAD), University of Cologne, Cologne, Germany; 5 German Center for Infection Research (DZIF), Partner Site Bonn-Cologne Department, Cologne, Germany; 6 Department of Infectious Diseases and Tropical Medicine, Heidelberg University Hospital, Heidelberg, Germany; 7 German Center for Infection Research (DZIF), Partner Site Heidelberg University Hospital, Heidelberg, Germany; 8 Internal Medicine V, Hematology, Oncology and Rheumatology, Heidelberg University Hospital, Heidelberg, Germany; 9 Department of Medicine III, Red Cross Hospital Munich, Munich, Germany; 10 Department of Internal Medicine II, University Hospital Würzburg, Würzburg, Germany; 11 Department of Medicine, Clinic III – Hematology, Oncology, Palliative Medicine, Rostock University Medical Center, Rostock, Germany; 12 Department of Internal Medicine II, Hematology, Oncology and Palliative Medicine, Asklepios Hospital Harz, Goslar, Germany; 13 Diagnostic and Interventional Radiology with Nuclear Medicine, Thoraxklinik, University of Heidelberg, Heidelberg, Germany; 14 Translational Lung Research Center (TLRC) at German Center for Lung Research (DZL), Heidelberg, Germany; 15 Diagnostic and Interventional Radiology, University Hospital Heidelberg, Heidelberg, Germany; 16 Department of Medicine, Clinic I - Hematology, Oncology, Infectiology, Department of Intensive Care and Emergency Medicine, Klinikum Bremen-Mitte, Bremen, Germany; 17 Department of Gastroenterology, Hepatology and Infectious Diseases, Medical Faculty and University Hospital Düsseldorf, Heinrich Heine University, Düsseldorf, Germany; 18 Department of Internal Medicine I, University Hospital Bonn, Bonn, Germany; 19 Center for Integrated Oncology (CIO), Aachen, Bonn, Cologne, Düsseldorf (ABCD), Partner-Site Bonn, Bonn, Germany; 20 Department of Oncology, Hematology, and Bone Marrow Transplantation with Section of Pneumology, University Medical Center Hamburg-Eppendorf, Hamburg, Germany; 21 Department of Hematology, Oncology and Cell Therapy, Medical Faculty, Otto von Guericke University Magdeburg, Magdeburg, Germany; 22 Department of Medicine III, University Hospital LMU Munich, Munich, Germany; 23 Institute of Virology, University Hospital Essen, University of Duisburg-Essen, Essen, Germany; 24 Institute for Diversity Medicine, Ruhr University Bochum, Bochum, Germany; 25 Hematology, Oncology, Stem Cell Transplantation and Cell Therapy, University Hospital Knappschaftskrankenhaus Bochum, Bochum, Germany; 26 Department of Hematology, Oncology and Palliative Care, St. Josef Hospital, Ruhr University Bochum, Bochum, Germany; 27 Hematology Oncology Germering, Germering, Germany; 28 Department of Intensiv Care Medicine, Agaplesion Diakonieklinikum Rotenburg, Rotenburg, Germany

**Keywords:** Respiratory virus, COVID-19, SARS-CoV-2, Influenza, Respiratory syncytial virus, Cancer, Solid tumor, Hematologic malignancy, Guideline

## Abstract

Community-acquired respiratory viruses (CARV), such as influenza-, parainfluenza- or respiratory syncytial virus, pose a significant threat to immunocompromised patients with cancer. Following the COVID-19 pandemic, SARS-CoV-2 has now joined the ranks of endemic respiratory viruses and continues to be a cause of significant morbidity and mortality in patients with cancer. Strategies to protect this vulnerable patient population both by prevention of infection and by early therapeutic intervention in case of infectious disease are therefore of utmost importance. This guideline provides updated evidence-based recommendations on diagnosis, prophylaxis and treatment of CARV infections including COVID-19 in patients with solid tumors or hematologic malignancies to support clinicians in offering optimal care. The guideline is based on a systematic review of currently available data and was developed until the beginning of 2025 by an expert panel of the Infectious Diseases Working Party (AGIHO) of the German Society for Hematology and Medical Oncology (DGHO).

## Introduction

Community-acquired respiratory virus (CARV) infections present a significant clinical challenge in cancer patients, especially in severely immunocompromised patients and patients undergoing hematopoietic stem cell transplantation (HCST) or following modern cellular therapies such as chimeric antigen receptor (CAR) T-cells. In recent years, SARS-CoV-2 has joined the arsenal of CARVs which comprise members of the orthomyxoviridae (influenza A, B, and C virus), the paramyxoviridae (human parainfluenza virus (hPIV) 1–4, and the pneumoviridae (human respiratory syncytical virus (RSV) A and B, and human metapneumovirus (hMPV). Furthermore, CARVs include members of the coronaviridae (human coronaviruses (hCoV)−229E, -HUK1, -OC43, and -NL63), the picornaviridae (human rhinovirus), the human adenoviridae (hAdV, with types mainly belonging to species B and C), as well as the polyomaviridae (KI polyomavirus and WU polyomavirus), and the parvoviridae (human bocavirus). While influenza virus and RSV infections adhere to seasonal peaks over the winter months [1, 2], parainfluenza virus, rhinovirus and SARS-CoV-2 infections pose a relevant infection risk year-round [3–5], affecting infection control practices on oncology wards and outpatient clinics as well as patients’ private life. Immunocompromised cancer patients may develop critical illness due to lower respiratory tract disease (LRTD) following CARV infection associated with significant morbidity and mortality, therefore this guideline presents updated information on diagnosis of CARV infection, impact on oncological treatment course, emerging treatment strategies and preventive measures.

## Methods

This guideline was developed in a formalized, step-by-step process by an expert panel from the Infectious Diseases Working Party (AGIHO) of the German Society for Hematology and Medical Oncology (DGHO). This panel consisted of 20 medical specialists certified in the fields of medical oncology, hematology, infectious diseases, critical care, emergency medicine, radiology, microbiology, and virology.

This guideline presents an update on our current guidelines on CARV infections [6] and on COVID-19 [7–9] in patients with cancer. The guideline process consisted of definition of topics by the expert panel, followed by a systematic search of relevant literature on MEDLINE for publications using one of the following search terms: “influenza”, “respiratory syncytial virus”, “SARS-CoV-2”, “COVID-19”, “parainfluenza”, “human metapneumovirus”, “rhinovirus”, “coronavirus”, “rhinovirus”, “vaccine/vaccination”, “prophylaxis/prevention”, or “therapy/treatment”. Publications were evaluated that appeared online until February 28th, 2025.

The relevant literature was collected, and a thorough review was performed, followed by extraction and rating of the data. After data analysis, preliminary recommendations were formulated, discussed and revised in a stepwise process by the expert panel. Grading of strength of recommendation and quality of evidence was performed applying the scale proposed by the European Society of Clinical Microbiology and Infectious Diseases (ESCMID) [10] (Table 1). The final recommendations presented in this guideline were discussed and agreed upon by the AGIHO general assembly.

**Table 1:** Grading system for strength of recommendation (SoR) and quality of evidence (QoE) as proposed by ESCMID

Strength of recommendation	
A	AGIHO strongly supports a recommendation for use
B	AGIHO moderately supports a recommendation for use
C	AGIHO marginally support a recommendation for use
D	AGIHO supports a recommendation against use
Quality of evidence	
I	Evidence from at least one properly designed randomized, controlled trial
II*	Evidence from at least one well-designed clinical trial, without randomization; from cohort- or case-control analytic studies (preferably from more than one centre); from multiple time series; or from dramatic results from uncontrolled experiments
III	Evidence from opinion of respected authorities, based on clinical experience, descriptive case studies, or report of expert committees
*added index for level II	
r	Meta-analysis or systematic review of randomized controlled trials
t	Transferred evidence, i.e. results from different patients’ cohorts or similar immune status situation
h	Comparator group is a historical control
u	Uncontrolled trial
a	Abstract published at an international meeting or manuscript available on preprint server only

### Clinical relevance and risk factors

The COVID-19 pandemic has underscored the substantial morbidity and mortality associated with CARV infections in immunocompromised patients with cancer [11, 12]. Although COVID-19-related mortality in the general population has markedly declined since the emergence of the omicron variant and its subvariants [13, 14], patients with cancer continue to face an increased risk of severe illness [15]. Moreover, even before the pandemic, non-SARS-CoV-2 respiratory viruses were a significant cause of morbidity and mortality in patients with cancer, a threat that persists to this day [16, 17].

Depending on the specific population of patients and CARV analysed, incidences of LRTD and mortality rates differ widely. Given the high worldwide interest, the level of available high quality study data is most solid for SARS-CoV-2 compared to other CARV. General risk factors for severe disease reported across various studies and CARV include, amongst others, hematologic malignancy, metastatic disease, active cancer, lymphopenia, hypogammaglobulinemia, and lack of antiviral therapy during upper respiratory tract infection [18–24]. Furthermore, co-infections with pathogens, in particular bacteria or fungi, are common and associated with adverse outcome [25, 26].

## Diagnosis of CARV infection

Prompt diagnosis of CARV infection in immunocompromised patients is of paramount importance in order to enable early antiviral therapy and to inform on infection control measures. A high level of awareness about possible CARV infections in symptomatic patients is therefore crucial and should consider local and current epidemiology.

Consistent with our previous guidelines [6–9], symptomatic patients with suspected CARV infection should be diagnosed using combined nasal and throat swabs, nasopharyngeal aspirates or in case of LRTD bronchoalveolar lavage fluid or tracheal aspirates (AIIt, Table 2 [27–30]),. Combination of multiple samples including saliva, sputum and oropharyngeal swabs may enhance the diagnostic yield. We recommend stringent testing of symptomatic patients for influenza viruses, RSV, hPIV and SARS-CoV-2 (AIIt [31–35]),. In severely immunocompromised patients (e.g. after allogeneic stem cell transplantation) with respiratory symptoms it may be reasonable to broaden the panel and to add testing for hMPV, hAdV, rhinovirus and non-SARS-CoV-2 hCoV (CIIt [36]),. In light of the local epidemiology and risk profiles of the respective patient population we recommend the implementation of institution-based testing guidelines.

**Table 2:** Recommendations on diagnosis of CARV infections in patients with cancer

Population	Intention	Intervention	SoR	QoE	Reference
symptomatic patients with cancer	to detect viral pathogen	combined nasal/throat swabs, nasopharyngeal aspirates or BAL fluid/tracheal aspirates	A	IItr	[27, 28, 30, 105]
symptomatic patients with cancer	to detect viral pathogen	NAT	A	IItr	[37]
symptomatic patients with cancer with respiratory symptoms at home	to detect SARS-Cov-2 and influenza infection	antigen test	C	IItu	[38–41]
immunosuppressed patients with cancer with known CARV infection	to detect viral shedding	repetitive testing	B	IIu	[51–54]
symptomatic patients with cancer	to detect LRTD in patient with CARV infection	chest X ray	D	III	[56]
symptomatic patients with cancer	to detect LRTD in patient with CARV infection	chest CT scan	A	III	[55, 59–61]

Regarding test modalities, nucleic acid amplification-based techniques (NAT) are most reliable in terms of sensitivity and specificity and are therefore highly recommended (AIItr [37]). Multiplex-PCR approaches, both inhouse and commercial, might reduce turnaround time and therefore time to diagnosis and should be used if available. Rapid antigen tests to detect SARS-CoV-2 or influenza virus can play a role for patients with respiratory symptoms at home as an initial assessment tool (CIItu [38–41]), but cannot replace NAT testing if patients need to be hospitalized. Therefore, in case of secondary hospital presentation, an additional NAT based test should be performed. Serology is not helpful in testing for acute infection and is hence discouraged (DIItr [42, 43]),.

Given the high vulnerability of immunocompromised patients, we strongly recommend respiratory panel testing as soon as even mild symptoms or signs occur (AIIu [44, 45]),. However, we do not see an indication for routine screening for CARV infections of asymptomatic patients outside an outbreak setting (DIII [45–47]),. In case of CARV infection, immunocompromised patients are at high risk for prolonged viral shedding [48]. Known risk factors are B-cell depletion and allogeneic HSCT [49, 50]. While this phenomenon has been described for several CARV, evidence is strongest for RSV and SARS-CoV-2 with viral shedding reported for > 100 days [51–53]. Although detection of viral RNA does not necessarily indicate clinically relevant infectivity, persistence of infectious SARS-CoV-2 proven by viral culture for weeks after initial diagnosis has been repeatedly demonstrated [53, 54]. We therefore recommend with moderate strength repetitive testing of immunosuppressed patients with known CARV infection to detect viral shedding (BIIu [53]), to guide ongoing infection control measures in hospitalized patients.

In patients with suspected viral LRTD, radiologic imaging can help to determine the degree of pulmonary involvement. To detect and characterize LRTD, a thin-section chest CT scan is the radiographic method of choice (AIIr [55–57]), while chest x-ray is discouraged in this setting due to its lack of sensitivity (DIIr [56, 58]),. Characteristic findings on CT scans are bronchial wall thickening indicative of tracheobronchitis and/or interstitial infiltrates in a ground-glass opacity consistent with interstitial thickening [59]. Ground-glass opacities (remained visibility of lung architecture such as intrapulmonary vessels) may be patchy or diffuse [59–61] and must be distinguished from consolidations, i.e., soft-tissue density, comparable to chest-wall muscle, which are typical of differential diagnoses including bacterial infection, especially with positive air bronchogram. Furthermore, the affection of the terminal bronchioles may manifest as “tree in bud” sign [60], bronchiolitis may further be visible as air-trapping, especially on image sets in expiration [59].

## Prevention

As outcome of CARV infection is often poor in immunocompromised patients, prevention by infection control measures and vaccination strategies are key and are further described in detail. Moreover, additional pharmacologic primary prophylaxis strategies, e.g., by monoclonal antibodies, may be discussed in individual patients, particularly with regard to SARS-CoV-2.

### Infection control measures

Much additional knowledge has been gained during the COVID-19 pandemic regarding efficacy as well as feasibility of different infection control measures aiming to reduce transmission of respiratory viruses. While a detailed discussion of all aspects of the various available infection control strategies is beyond the scope of this guideline, we highlight essential aspects. The following recommendations address the routine clinical settings in cancer care (Table 3).

**Table 3:** Recommendations on infection control measures and cancer care in the setting of CARV infections in patients with cancer

Population	Intention	Intervention	SoR	QoE	Reference
immunocompromised patients with cancer, close contacts and medical personnel	to prevent transmission	hand hygiene	A	IIt	[62, 63]
immunocompromised patients with cancer, close contacts and medical personnel	to prevent transmission	prefer face mask vs. no mask	A	IIt	[62–67]
immunocompromised patients with cancer, close contacts and medical personnel	to prevent transmission	prefer FFP2/N95 vs. surgical mask	B	IItr	[68–70]
immunocompromised patients with cancer with CARV and their contact patients	to prevent transmission	contact isolation	A	IIu	[71]
allo-HSCT/CAR-T and evidence of CARV, symptomatic infection	to prevent disease progression and improve survival	delay conditioning if possible	B	IIu	[47, 97]
all other chemo-/immuno-/targeted therapies and evidence of CARV, symptomatic infection	to prevent disease progression and improve survival	delay therapy, if possible	C	III	[98]
allo-HSCT/CAR-T and evidence of CARV, asymptomatic shedding	to prevent disease progression and improve survival	delay conditioning, if possible	C	IIu	[46, 47, 97]
all other chemo-/immuno-/targeted therapies and evidence of CARV, asymptomatic shedding	to prevent disease progression and improve survival	continue therapy	B	III	[98]
immunocompromised patients with cancer	to prevent influenza infection and improve survival	vaccination against Influenza	A	IIu	[72–76]
immunocompromised patients with cancer	to prevent SARS-CoV-2 infection and improve survival	vaccination against SARS-Cov-2	A	IIt	[77]
immunocompromised patients with cancer	to prevent RSV infection and improve survival	vaccination against RSV	A	IIt	[78–80]
immunocompromised patients with cancer without adequate vaccine protection	to prevent symptomatic SARS-CoV-2 infection	pre-exposure prophylaxis with sipavibart or pemivibart	C	IIt	[90, 91]

Thorough hand disinfection is a cornerstone of infection control management and is strongly recommended for patients, close contacts and medical personnel to prevent transmission of CARV (AIIt [62, 63]),. The efficacy of face masks to reduce both the viral load in expiratory breaths as well as to reduce the overall infection rates has been repeatedly shown [62–67]. We therefore strongly recommend the use of face masks to prevent transmission of CARV in patients with cancer (AIIt [62–67]),. Filtering Face Piece (FFP) 2 masks and their US American counterparts N95 respirators are characterized by a closer fit and finer mesh and have been associated with a further reduction in virus transmissions and viral infections compared to surgical masks in two large meta-analyses [68–70]. Especially for high-risk contacts as well as high-risk interventions (e.g., bronchoscopy) FFP2 masks/N95 respirators are therefore preferable to surgical face masks with a moderate strength of recommendation (BIItr [68, 69]),. In case of CARV infections in hospitalized patients with cancer, isolation of contact patients should be established in order to prevent further transmission and nosocomial outbreaks (AIIu [71]).

Emphasis lies on developing and adhering to local infection control protocols including comprehensive outbreak prevention and management strategies that consider local epidemiology.

### Vaccination

Active immunization by vaccination is next to infection control strategies one of the most important preventive measures against CARV. Currently, CARV vaccines are available and approved by both the European Medicines Agency (EMA) and the United States Food and Drug Administration (FDA) against COVID-19, influenza, and RSV.

Data on vaccination in patients with cancer is predominantly based on smaller observational studies for most CARV vaccinations. However, there is nowadays a solid body of evidence in support of influenza vaccination [72–76] and the clinical benefit of vaccination against SARS-CoV-2 has been outstanding in this patient cohort [77]. Recent data on vaccination against RSV have also shown a high effectiveness in the prevention of severe disease with a favorable safety profile [78–80]. Although data on effectiveness of RSV vaccination in patients with cancer has yet to be published.

Regarding vaccination of patients with cancer, special attention should be drawn to highly immunocompromised patients, e.g., with lymphatic disorders and/or B-cell depletion following anti-CD20- or anti-CD38-antibody treatment, in whom antibody titers, as a surrogate for protection, are not seen equivalently to immunocompetent people [81, 82]. Still, in these patients, T-cell responses can be elicited, as recently shown with vaccinations against SARS-CoV-2 [83]. It should also be noted that live vaccines are largely contraindicated (DIII [84]), as they can cause serious infections in immunocompromised patients [85]. With regard to vaccines against CARV this currently applies to the intranasal live vaccine against influenza [86].

Consistent with our previous guidelines, seasonal vaccination against influenza and SARS-CoV-2 is strongly recommended for all patients with cancer and their close contacts including medical personnel (AIIu [7, 87], Table 3). Vaccination against RSV is strongly recommended for patients with cancer who can be considered immunocompromised either due to an ongoing hematologic malignancy, e.g., chronic lymphocytic leukaemia or multiple myeloma, or due to a recent immunocompromising antineoplastic therapy or who are scheduled to receive such therapy (AIIt [78–80]). For more detailed recommendations on vaccination schedules and specific patient subgroups we refer to the current guideline on vaccination in patients with cancer [6].

### Passive immunization

In patients without adequate vaccine response, passive immunization with the monoclonal antibody combination tixagevimab/cilgavimab has shown to significantly reduce the rate of COVID-19 in high-risk patients in the phase-III PROVENT trial [88]. However, these and other monoclonal antibodies licensed in the pandemic era for pre- or post-exposure prophylaxis against COVID-19 no longer retain adequate neutralizing activity at the writing of this guideline against predominant variants [89] and can therefore currently not be recommended. Adapted antibodies such as e.g., pemivibart and sipavibart, were evaluated in the post-pandemic era in the randomized phase III SUPERNOVA and CANOPY trials, respectively, showing moderate efficacy as pre-exposure prophylaxis [90, 91]. In vitro data on neutralizing efficacy of sipavibart and pemivibart against various JN.1 sublineages showed mixed results depending on the respective subvariant [92], in particular, spike protein mutation F456L has been identified as conferring resistance to sipavibart [93]. This highlights the need to closely monitor local variant landscape. At the writing of this guideline in mid 2025 F456L variants accounted for > 90% of cases in Europe [94]. Hence, pre-exposure prophylaxis with pemivibart or sipavibart cannot be recommended in view of the predominant F456L variants currently in circulation (DIIt [90, 91]).

Administration of polyvalent intravenous immunoglobulin preparations (IVIGs) is approved by EMA for patients with significant hypogammaglobulinemia (immunoglobulin G < 400 mg/dl) and recurrent infections or inadequate vaccine response as a prophylaxis strategy. The current rise in CAR-T-cell therapies and treatments with bispecific antibodies which often cause prolonged and severe hypogammaglobulinemia has rendered this topic more important and recent guidelines support stringent IVIG prophylaxis for many of these patients to ensure immunoglobulin G levels > 400 mg/dl [95, 96]. However, there is currently insufficient data on the specific role of IVIG prophylaxis against CARV infections. Therefore, no specific recommendation can be made on its use as well as on timing of administration outside of the approved indications.

## Cancer care in the setting of CARV infection

CARV infection often interferes with oncologic treatment schedules. In patients scheduled for allogeneic HSCT or CAR-T-cell therapy with CARV infection, conditioning therapy should be delayed if possible due to the significant risk of severe viral pneumonia (BIIu, Table 3 [47, 97]). In oligosymptomatic patients requiring timely therapy initiation, adapted management strategies based on stringent risk-benefit analysis may be employed. In patients with known asymptomatic CARV shedding, conditioning treatment for allogeneic HSCT or CAR-T-cell therapy may be postponed (CIIu [46, 47, 97]), however, testing strategies vary across transplantation centers and outcomes of patients with asymptomatic CARV infection undergoing allogeneic SCT or CAR-T-cell therapy have not been systematically assessed.

In all other patients with symptomatic CARV infection undergoing chemo- or immunotherapy, delaying therapy can be beneficial for the subsequent course of CARV disease or for disease progression (**CIII** [98]). Nevertheless, in patients in need of urgent chemotherapy, treatment delays need to be critically discussed and modified management is recommended, especially in patients with oligosymptomatic CARV disease. In contrast, for patients tested positive for CARV infection with asymptomatic shedding, oncologic treatment should be continued (BIII [98]), especially in patients with long-term asymptomatic SARS-CoV-2 shedding [99, 100]. In these patients, antiviral therapy should only be reinitiated in case of a new course of symptomatic infection.

## Treatment of CARV infection

Immunocompromised patients are at significant risk of severe disease by CARV. Directly acting antivirals should be offered to patients whenever such agents are available. As a general guiding principal, it is strongly recommended to initiate antiviral therapy as early as possible after symptom onset and diagnosis of CARV infection to prevent disease progression and reduce mortality (AIIt, Table 4). Data supporting this paradigm has predominantly been generated in the setting of influenza or COVID-19. A large retrospective study in patients with solid tumours and influenza showed a significantly reduced mortality if antiviral treatment with neuraminidase inhibitors was initiated within 48 h of symptom onset [101]. Furthermore, treatment initiation at the stage of upper respiratory tract infection significantly reduced the rate of progression to LRTD [101]. Regarding COVID-19, large phase III studies on remdesivir, molnupiravir or nirmatrelvir/ritonavir as early out-patient therapy all required treatment initiation within a time frame of 5–7 days following symptom onset/diagnosis [102–104]. In severely immunocompromised patients, a delayed diagnosis of CARV infection should not preclude antiviral therapy.

**Table 4:** Recommendations on treatment of CARV infections in patients with cancer: influenza, PIV, RSV, and adenovirus

Population	Intention	Intervention	SoR	QoE	Reference
immunocompromised patients with cancer with symptomatic CARV infection	to prevent LRTID and reduce mortality	start antiviral therapy as soon as possible	A	IIt	[101–104, 106]
immunocompromised patients with cancer with influenza	to shorten duration and prevent LRTID	oseltamivir	A	IIt	[31, 106–108, 110]
immunocompromised patients with cancer with influenza	to shorten duration and prevent LRTID	zanamivir	A	IIt	[109, 110]
immunocompromised patients with cancer with influenza	to shorten duration and prevent LRTID	baloxavir	B	IIt	[111]
immunocompromised patients with cancer with influenza	to prevent LRTID and improve survival	IVIG	C	III	[107]
immunocompromised patients with cancer with RSV	to prevent LRTID and improve survival	ribavirin	B	IIu	[22, 23, 33, 118, 120–122]
immunocompromised patients with cancer with RSV	to prevent LRTID and improve survival	IVIG	B	IIu	[33, 123, 124]
immunocompromised patients with cancer with RSV	to prevent LRTID and improve survival	palivizumab	C	IItu	[33, 125]
immunocompromised patients with cancer with parainfluenza	to prevent LRTID and improve survival	ribavirin	C	III	[24, 34, 127]
immunocompromised patients with cancer with parainfluenza	to prevent LRTID and improve survival	IVIG	C	III	[26]
immunocompromised patients with cancer with adenovirus-associated pneumonitis	to cure	cidofovir	B	IItu	[128, 129]

Treatment with antibacterial agents is obviously not causative against viral infections and is discouraged in the absence of suspected or proven bacterial co-infection (DIItr [105]). However, as mentioned previously, bacterial or fungal co-infections are a frequent and significant risk factor for adverse outcome in patients with cancer and CARV infection and should be treated accordingly (AIItr [25, 26]).

### Influenza

Immunocompromised patients with influenza should receive antiviral treatment as early as possible, ideally within 48 h of symptom onset [106]. While risk of progression to severe disease varies between patients with cancer, those with hematologic malignancy, metastatic disease or receiving immunosuppressive anticancer therapies should be considered high-risk and treated with neuraminidase inhibitors routinely in case of influenza virus infection [32, 107, 108]. Treatment with neuraminidase inhibitors oseltamivir and zanamivir reduced risk of progression to LRTD and complicated courses in retrospective studies in patients with solid tumors, hematologic malignancies or allogeneic HSCT [31, 109, 110]. We therefore recommend treatment with oseltamivir and zanamivir with moderate strength in patients with cancer and influenza virus infection to shorten duration of infection and to prevent LRTD (BIIt, Table 4). Baloxavir has a favourable safety profile in renal insufficiency and has few drug-drug interactions, however, it was not studied in patients with cancer and is therefore only recommended with marginal strength (CIIt [111]),. A prolonged treatment course might be reasonable especially in severely immunocompromised patients. In hypogammaglobulinemia of IgG < 4 g/L, intravenous immunoglobulins (IVIGs) can be considered as a supportive treatment measure (CIII [107]).

Neuraminidase inhibitors have also been evaluated as post-exposure prophylaxis both in high-risk settings (e.g. patients undergoing allogeneic stem cell transplantation) [112] as well as in standard-risk patients without a cancer diagnosis [113]. A current meta-analysis based on 33 RCTs exploring six antivirals in post-exposure prophylaxis underscores the importance of post-exposure prophylaxis to decrease the risk of influenza in high-risk patients [114]. We therefore recommend to administer post-exposure prophylaxis promptly after exposure (ideally within 72 h) in immunocompromised patients with cancer (BIIt).

### RSV

RSV has been increasingly recognized as a cause for significant morbidity and mortality in high-risk adults such as immunocompromised patients with cancer [16, 21]. We therefore recommend to initiate antiviral therapy in immunocompromised patients with symptomatic RSV infection as soon as possible, ideally at the stage of upper respiratory tract infection to prevent progression to LRTD and reduce mortality. While data is most robust for allogeneic HSCT recipients [22, 33], several studies support benefit of antiviral treatment also in less immunocompromised patients with cancer [23, 115]. Of note, some authors report a favourable course of RSV infection even in immunocompromised patients, such as autologous HSCT recipients, without specific therapeutic intervention [116, 117].

Treatment algorithms of RSV infections in adult patients with cancer are based on administration of antiviral therapy with ribavirin and IVIGs. Ribavirin is the mainstay of RSV treatment since it has been shown to reduce progression to LRTD and improve survival [22, 23, 118]. While data is most robust for aerosolized ribavirin [22, 33], its administration is cumbersome, expensive and potentially teratogenic [119] and has therefore mostly been abandoned in routine clinical care. Oral or intravenous administration of ribavirin has been repeatedly shown to be an effective antiviral treatment option in patients with cancer with RSV infection [23, 33, 120, 121], despite not being licensed for this indication by FDA nor EMA. Recently, a large retrospective study on HSCT patients with RSV infections showed comparable survival rates of aerosolized and oral ribavirin [122]. We recommend antiviral treatment with ribavirin in immunocompromised patients with cancer and symptomatic RSV infection with moderate strength (BIIu, Table 4).

Administration of IVIGs in combination with antiviral therapy can provide additional benefit in immunocompromised patients with cancer and is therefore recommended with moderate strength (BIIu [33, 121, 123, 124]). There is currently insufficient data to support that high-titer RSV immunoglobulin is more effective than standard immunoglobulin preparations [33, 124]. Palivizumab, a monoclonal antibody binding the A epitope of the RSV fusion protein, is licenced by EMA and FDA for prevention of RSV disease in children at high risk, while data in adult cancer patients is limited [33, 125]. We recommend palivizumab with marginal strength in severely immunocompromised adult patients with cancer with RSV disease (CIItu [33, 125]). Nirsevimab is licensed for prevention of RSV LRTD in newborns, however we did not find data in adult patients with cancer.

### Parainfluenza

While hPIV infections have been associated with significant morbidity and mortality in patients with cancer [126], evidence for effective antiviral strategies is scarce [34, 127]. A large retrospective analysis of allogeneic HSCT recipients with hPIV infections suggested a possible benefit of treatment with ribavirin reducing progression to LRTD, however, the impact on mortality is not yet clear [24]. Therefore, treatment with ribavirin may be considered in immunocompromised patients with hPIV infection, but is only recommended with marginal strength (CIII, Table 4). Similarly, administration of IVIGs may be a possible supportive measure, albeit with low quality of evidence and marginal strength of recommendation (CIII [26]).

### Adenovirus

In human adenovirus infections, antiviral treatment with cidofovir may alleviate disease course and improve viral clearance in immunocompromised patients with cancer [128, 129] and is therefore recommended with moderate strength (BIItu, Table 4). Cidofovir can either be applied at 5 mg/kg weekly or, in patients with reduced kidney function, at 1 mg/kg thrice weekly for two weeks, followed by a week off. In the allogeneic HSCT setting, individualized strategies like donor-lymphocyte infusion [130] or adoptive transfer of specific T cells [131] may be considered.

### hMPV, rhino- and coronaviruses (non-SARS-CoV-2)

Currently, no convincing evidence for effective antiviral strategies against hMPV, rhinovirus or non-SARS-CoV-2 coronavirus infections is available. Ribavirin has been evaluated as an antiviral treatment strategy in hMPV infections in allogeneic HSCT recipients, but no clear benefit was demonstrated [132]. Therefore, no treatment recommendations can be made for infection with hMPV, rhino- and non-SARS-CoV-2 coronaviruses.

### SARS-CoV-2 (COVID-19)

While overall mortality due to COVID-19 has decreased in the general population with the advent of vaccinations, increased population immunity and emergence of novel SARS-CoV-2 omicron sub-variants, immunocompromised patients with cancer remain at significant risk of COVID-19-associated morbidity and mortality [133].

Early antiviral therapy is crucial to improve outcomes in immunocompromised patients with COVID-19 [134]. In the multi-center EPICOVIDEHA registry study on patients with hematologic malignancies and COVID-19, treatment with nirmatrelvir/ritonavir was associated with lower mortality compared to other targeted drugs [135]. We strongly recommend early antiviral therapy with nirmatrelvir/ritonavir as soon as symptoms develop, ideally within 72 h of symptom onset, to prevent disease progression, hospitalization and/or death in non-hospitalized cancer patients (AIIt, Table 5 [104, 135–138]). While the pivotal trial on nirmatrelvir/ritonavir was performed in non-hospitalized patients [139], cancer patients may acquire COVID-19 while hospitalized for other reasons and similarly benefit from early antiviral therapy. We therefore think that data can be transferred to the setting of cancer patients with mild COVID-19 hospitalized for other reasons. As ritonavir is a strong inhibitor of CYP3A4, it is important to assess any drug-drug interactions, in particular for patients on active anti-cancer treatment, e.g., venetoclax, neratinib, apalutamid, or receiving immunosuppressive medication. A comprehensive checklist of interactions can be found e.g. at https://www.covid19-druginteractions.org/checker. If treatment with nirmatrelvir/ritonavir is not possible, we strongly recommend early antiviral therapy with remdesivir as a three-day course (AIIt [102]), based on the results of the phase-III PINETREE study. Molnupiravir was widely used mid-pandemic as early antiviral therapy, however, it is no longer approved in Europe as final analysis of its pivotal trial did not match a more promising interim analysis [103]. In patients with hematologic malignancies, a matched-pair analysis of the EPICOVIDEHA registry reported a comparable efficacy of molnupiravir to nirmatrelvir/ritonavir allowing for a recommendation with marginal strength (CIIt [137]).

**Table 5:** Recommendations on treatment of CARV infections in patients with cancer: COVID-19

Population	Intention	Intervention	SoR	QoE	Reference
patients with cancer with COVID-19 - ambulatory, mild	to prevent hospitalization and/or death	nirmatrelvir/ritonavir	A	IIt	[104, 135–137]
patients with cancer with COVID-19 - ambulatory, mild	to prevent hospitalization and/or death	remdesivir	A	IIt	[102]
patients with cancer with COVID-19 - ambulatory, mild	to prevent hospitalization and/or death	molnupiravir	C	IIt	[137]
patients with cancer with COVID-19 - ambulatory, mild and hospitalized, mild to moderate	to accelerate viral clearance and to shorten course of disease	combination treatment remdesivir with nirmatrelvir/ritonavir	C	IIu	[140–143]
patients with cancer with COVID-19 - hospitalized, moderate (no oxygen therapy, low-flow oxygen)	to shorten time to recovery	remdesivir for 5–10 days	B	IIt	[145–148]
patients with cancer with COVID-19 - hospitalized, moderate (low-flow oxygen)	to reduce mortality	dexamethasone 6 mg/d for up to 10 days	A	IIt	[150]
patients with cancer with COVID-19 - hospitalized, moderate (low-flow oxygen) and systemic inflammation	to reduce mortality	JAK inhibitors	B	IIt	[151, 152, 158–160]
patients with cancer with COVID-19 - hospitalized, moderate (low-flow oxygen) and systemic inflammation	to reduce mortality	tocilizumab	B	IIt	[150, 164]
patients with cancer with COVID-19 - hospitalized, moderate (low-flow oxygen) and systemic inflammation	to reduce mortality	anakinra	C	IIt	[165, 166]
patients with cancer with COVID-19 - hospitalized, all patients	to prevent thromboembolic complications	low-dose LMWH	A	IIt	[169, 170]
patients with cancer with COVID-19 - hospitalized, moderate plus additional risk factors*	to prevent thromboembolic complications and reduce mortality	intermediate-dose or therapeutic anticoagulation	B	IIt	[169, 171–174]
patients with cancer with COVID-19 – hospitalized, severe (high-flow oxygen/NIV, MV, ECMO)	to prevent thromboembolic complications and reduce mortality	intermediate-dose anticoagulation	B	IIt	[171]
patients with cancer with COVID-19 - hospitalized, severe (high-flow oxygen/NIV, MV, ECMO)	to prevent thromboembolic complications and reduce mortality	routine therapeutic anticoagulation	D	IIt	[171, 172, 175]

*significantly elevated D-dimer levels and/or additional risk factors and/or prior thromboembolic complications

Patients with severe immunosuppression are at risk of prolonged or relapsing COVID-19 [53, 54]. Several case series or retrospective studies reported high rates of viral clearance when combining nirmatrelvir/ritonavir and remdesivir with or without monoclonal antibodies [140–143]. Combination of nirmatrelvir/ritonavir and remdesivir may be considered to induce viral clearance and to shorten the course of disease (CIIu [140–143]). Regarding prolonged therapy with antivirals, a recent phase II trial evaluating 5 vs. 10 vs. 15 day regimens of nirmatrelvir/ritonavir in non-hospitalized immunocompromised patients with COVID-19 did not find a significant difference in outcome between treatment durations, but good tolerance [144]. Therefore, prolonged antiviral treatment cannot currently be recommended outside of exceptional cases.

Several monoclonal antibodies have been approved for treatment of COVID-19, but none of the approved monoclonal antibodies retained sufficient neutralizing activity against SARS-CoV-2 variants predominant at the time of writing. Therefore, no recommendations are made on monoclonal antibody treatment.

In hospitalized patients with moderate symptoms of COVID-19, remdesivir has shortened time to recovery [145–148]. Greatest benefit was observed in patients receiving low-flow oxygen. We therefore recommend treatment with remdesivir for 5–10 days in hospitalized patients with cancer and moderate COVID-19 with moderate strength (BIIt [145–149]). Immunomodulatory treatment, e.g., dexamethasone, tocilizumab, sarilumab, baricitinib, is not indicated in patients without need of oxygen support (DIIt [150–152]. Treatment with convalescent plasma has been abandoned in routine clinical care and is discouraged in seropositive patients [153, 154]. In seronegative moderately ill patients at high risk for adverse outcomes, convalescent plasma may be considered on an individual basis in light of the RECOVER trial findings [155].

In patients with COVID-19 requiring oxygen therapy and showing signs of systemic inflammation, immunomodulatory treatment is essential to control SARS-CoV-2-induced hyperinflammation [156]. While early data available during the height of the pandemic and early guideline versions have underlined the importance of dexamethasone as first-line immunomodulatory treatment, recent meta-analyses of available RCTs and guideline updates have taken JAK inhibitors to the forefront [157–159]. JAK inhibition, specifically baricitinib, was shown to provide a consistent survival benefit with large effect size in three RCTs (2659 patients) and was associated with a low risk of adverse events [151, 152, 160]. Recent meta-analysis of the available RCTs proclaim that baricitinib reduced mortality on all level of respiratory support, independent of concomitant dexamethasone or tocilizumab treatment [159]. Hence, baricitinib is now recommended in patients with severe COVID-19 as first-line immunomodulatory treatment [157, 159] (BIIt) alongside with dexamethasone (AIIt) [150]. This recommendation is consistent with the current NIH guidelines [161]. Dexamethasone significantly reduced mortality in patients with COVID-19 requiring oxygen support in the RECOVERY trial [150]. Treatment with dexamethasone is strongly recommended in these patients (AIIt [150]), at a dose of 6 mg/day for up to 10 days or hospital discharge (whichever comes first). With regard to superiority of baricitinib in treating severe COVID-19 both in terms of effect size and side effects (e.g. lower rate of secondary infections) across the available RCTs [160, 162, 163], addition of anti-IL6R antibody tocilizumab (BIIt [164]) or anakinra (CIIt [165, 166]) are now considered alternative options in case of unavailability or contraindications to JAK inhibition [157, 158, 161]. Addition of anti-IL6R monoclonal antibody tocilizumab to treatment with dexamethasone reduced the risk of intubation and death in the RECOVERY trial [164]. In the CORIMMUNO trial the IL1 receptor antagonist anakinra failed to reduce the mortality rate in moderately severe COVID-19 [165], while the SAVE-MORE trial found a decreased mortality rate and shortened hospital stays [166]. On a more general note, pre-existing immunocompromise by cancer or its treatments needs to be considered when deciding on immunomodulatory therapies in patients with cancer and COVID-19. A retrospective analysis of the EPICOVIDEHA registry in patients with hematologic malignancies and COVID-19 suggested possible adverse effects of dexamethasone, particularly if patients did not receive antiviral therapies [167], thus highlighting the critical importance of antiviral treatment in severely immunocompromised patients, even later in the course of COVID-19.

Therapeutic algorithms for severe COVID-19 requiring mechanical ventilation or extra-corporeal membrane oxygenation (ECMO) generally follow those for non-cancer patients. We therefore refer to the relevant intensive care guidelines [168].

The high incidence of thromboembolic events in hospitalized patients with cancer and COVID-19 underscores the importance of thromboprophylaxis [169, 170]. All hospitalized patients with COVID-19 should receive prophylactic low-dose low molecular weight heparin except in the presence of individual contraindications (AIIt [169, 170]). In hospitalized moderately ill cancer patients with significantly elevated D-dimer levels and/or additional risk factors and/or prior thromboembolic complications, intermediate-dose or therapeutic anticoagulation should be considered when no increased risk of bleeding is present (BIIt [169, 171–174]). If these patients progress to severe COVID-19 pneumonia requiring intensive care management including high-flow oxygen, mechanical ventilation or ECMO treatment, the anticoagulation dosage should be adapted to the patient’s individual bleeding risk. Therapeutic anticoagulation in severely ill patients is discouraged unless venous thromboembolism is confirmed (DIIt [171, 172, 175]), though intermediate-dose anticoagulation can be considered in patients with low bleeding risk according to the results of the French ANTICOVID trial (BIIt) [171].

## Conclusion and outlook

Patients with cancer are a population at significant risk of morbidity and mortality due to CARV infections. High awareness, prompt diagnosis and early antiviral therapy are essential in protecting these vulnerable patients. Development of vaccines and optimal vaccination strategies in immunocompromised patients will further add to lower disease burden and improve outcomes.

## Data Availability

No datasets were generated or analysed during the current study.
